# P-1203. Gepotidacin activity against Escherichia coli and Klebsiella pneumoniae, including molecularly characterized ESBL- and carbapenemase-positive subsets causing urinary tract infections in United States Medical Centers (2023)

**DOI:** 10.1093/ofid/ofaf695.1396

**Published:** 2026-01-11

**Authors:** Rodrigo E Mendes, Zachary Kockler, Gina Morgan, Renuka Kapoor, Nicole E Scangarella-Oman, S J Ryan Arends

**Affiliations:** Element Iowa City (JMI Laboratories), North Liberty, IA; Element Iowa City (JMI Laboratories), North Liberty, IA; Element Iowa City (JMI Laboratories), North Liberty, IA; GSK, Atlanta, Georgia; GlaxoSmithKline plc., Collegeville, PA; Element Iowa City (JMI Laboratories), North Liberty, IA

## Abstract

**Background:**

Gepotidacin (GEP) was recently approved by the United States (US) Food and Drug Administration (FDA) for the treatment of uncomplicated urinary tract infections (uUTI). GEP is a novel, bactericidal, first-in-class triazaacenaphthylene antibiotic that inhibits bacterial DNA replication by a distinct binding site, a unique mechanism of action and provides well-balanced inhibition of two type II topoisomerases (for most pathogens). This study reports the activity of GEP and other oral agents against *E. coli* (EC) and *K. pneumoniae* (KPN), including ESBL- and carbapenemase-positive isolates.

**Figure:**
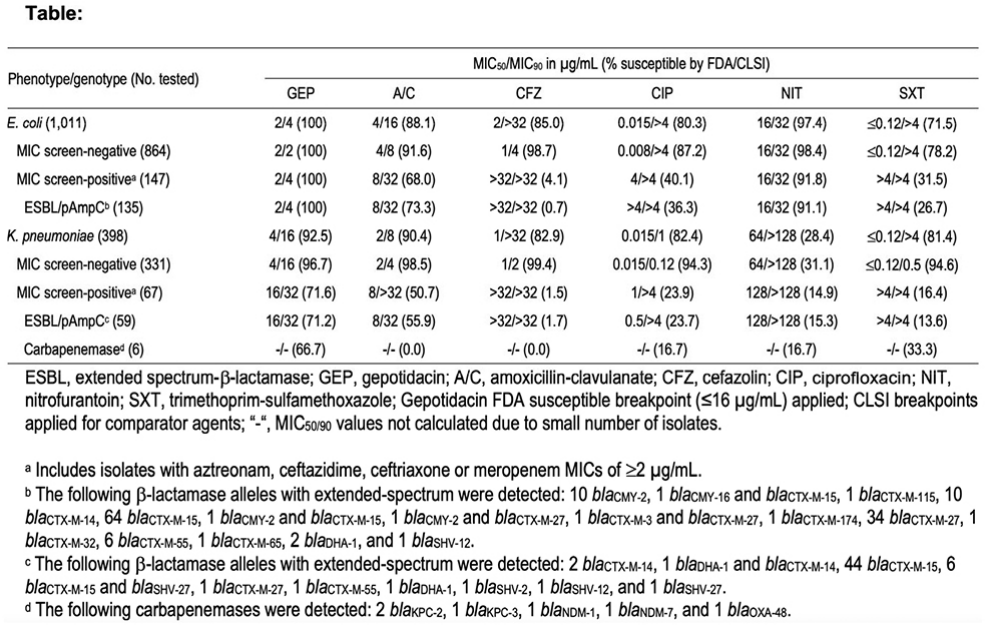

**Methods:**

1,409 isolates from 58 US sites were included. CLSI methods were used for susceptibility testing and MIC interpretation, except for GEP that used FDA breakpoints. Isolates with aztreonam, ceftazidime, ceftriaxone or meropenem MIC of ≥2 µg/mL were sequenced, and screened for plasmid-mediated AmpC (pAmpC), ESBL, and carbapenemase genes.

**Results:**

14.5% (147/1,011) of EC met the MIC criteria and screened for β-lactamase genes. Among this EC subset, 91.8% (135/147) carried ESBL and/or pAmpC genes. GEP had MIC_50/90_ of 2/4 µg/mL against isolates that met the MIC criteria for β-lactamase screening and the subset carrying ESBL and/or pAmpC genes. In addition, GEP inhibited all EC (100%S) at the FDA susceptible (S) breakpoint of ≤16 µg/mL, regardless of resistance (R) phenotype or genotype. Only nitrofurantoin showed activity (91.1–91.8%S) against these EC subsets. 16.8% (67/398) KPN met the MIC criteria and were screened for β-lactamase genes. Among this subset, 88.1% (59/67) carried ESBL/pAmpC genes, whereas 9.0% (6/67) carried carbapenemases. GEP (MIC_50/90_, 4/16 µg/mL) was active against KPN (92.5%S). GEP was active against 96.7% of KPN that did not meet the MIC criteria for β-lactamase screening, and 71.6% of those that met the MIC criteria. Oral comparators showed S ≤50.7% against the latter KPN group.

**Conclusion:**

In general, GEP showed S higher than oral comparator agents when tested against EC and KPN causing UTI in US hospitals. These data benchmark GEP against EC and KPN for subsequent monitoring after the recent FDA approval for the treatment of uUTIs.

**Disclosures:**

Rodrigo E. Mendes, PhD, GSK: Grant/Research Support|Shionogi & Co., Ltd.: Grant/Research Support|United States Food and Drug Administration: FDA Contract Number: 75F40123C00140 Renuka Kapoor, PhD, GSK: Employee|GSK: Stocks/Bonds (Public Company) Nicole E. Scangarella-Oman, MS, GSK: Employee|GSK: Stocks/Bonds (Public Company)

